# Facile Synthesis of Mono-Dispersed Polystyrene (PS)/Ag Composite Microspheres via Modified Chemical Reduction

**DOI:** 10.3390/ma6125625

**Published:** 2013-12-04

**Authors:** Wen Zhu, Yuanyuan Wu, Changhao Yan, Chengyin Wang, Ming Zhang, Zhonglian Wu

**Affiliations:** 1 College of Chemistry and Chemical Engineering, Yangzhou University, Yangzhou 225002, China; E-Mails: czzwen@163.com (W.Z.); 1747168992@qq.com (Y.W.); 250178276@qq.com (C.Y.); 2 College of Materials Science and Engineering, Jiangsu University of Technology, Changzhou 213001, China; E-Mail: 871765383@qq.com (Z.W.)

**Keywords:** silver nanoparticle, PS/Ag composite, dopamine, PVP, hollow microsphere

## Abstract

A modified method based on *in situ* chemical reduction was developed to prepare mono-dispersed polystyrene/silver (PS/Ag) composite microspheres. In this approach; mono-dispersed PS microspheres were synthesized through dispersion polymerization using poly-vinylpyrrolidone (PVP) as a dispersant at first. Then, poly-dopamine (PDA) was fabricated to functionally modify the surfaces of PS microspheres. With the addition of [Ag(NH_3_)_2_]^+^ to the PS dispersion, [Ag(NH_3_)_2_]^+^ complex ions were absorbed and reduced to silver nanoparticles on the surfaces of PS-PDA microspheres to form PS/Ag composite microspheres. PVP acted both as a solvent of the metallic precursor and as a reducing agent. PDA also acted both as a chemical protocol to immobilize the silver nanoparticles at the PS surface and as a reducing agent. Therefore, no additional reducing agents were needed. The resulting composite microspheres were characterized by TEM, field emission scanning electron microscopy (FESEM), energy-dispersive X-ray spectroscopy (EDS), XRD, UV-Vis and surface-enhanced Raman spectroscopy (SERS). The results showed that Ag nanoparticles (NPs) were homogeneously immobilized onto the PS microspheres’ surface in the presence of PDA and PVP. PS/Ag composite microspheres were well formed with a uniform and compact shell layer and were adjustable in terms of their optical property.

## 1. Introduction

As shown in recent studies, various methods have been developed to expand the application area of noble metal nanoparticles and to control the morphology and the behavior. Composite microspheres with noble metal nanoshells have great advantages in catalysis, optics, conductivity, chemical sensors, and so on [1,2,3,4,5]. Moreover, these hybrid materials can prevent noble metal nanoparticles from agglomerating without the use of a stabilizer and can be easily retrieved, owing to the relatively large size of the supports. In particular, silver, with its outstanding combination of properties, has continued to be of great interest in terms of its potential applications in composite materials.

To date, considerable efforts have been put in to integrate noble metals into support particles. One approach is to coat silica or polystyrene with silver nanoshells. The prepared methods can be divided into two main categories [6,7]. The first one is described as follows. Template particles are treated by some chemicals or a chemical process to make the surfaces of the template particles bear functional groups. Then, the precursors of noble metals are added to the template particles. By adding the reducing agent, the precursors are reduced to zero-valent metals. Thus, the composite microspheres are obtained. The second process, called the layer-by-layer (LBL) self-assembly technique, is also widely used to prepare noble metal composite microspheres [8,9]. Mayer *et al.* prepared polystyrene/silver composite microspheres through an *in situ* reduction method [10], but this approach had shortcomings, such as incomplete coverage, rough surfaces, nonuniformity in shell thickness and aggregation of polystyrene (PS)/Ag microspheres. Another promising approach is to fabricate composite microspheres containing homogeneously dispersed metal nanoparticles. Wang and Asher had prepared silica spheres containing dispersed silver particles in a micro-emulsion [11]. However, it is difficult to control the diameter in a wide range, and the size distribution is often too wide. Therefore, improving the traditional technology of preparing mono-dispersed composite microspheres with uniform and complete Ag nanoshells is urgently needed.

In this work, a facile method for preparing PS/Ag composite microspheres was presented. The emphasis of this work was on the control of the monodispersity, shell thickness, average density and regularity in the morphology of PS/Ag composite microspheres. The poly-vinylpyrrolidone (PVP) and poly-dopamine (PDA) with hydroxyl and amino groups played an extremely important role, anchoring silver ions into the PS matrix. On the basis of the experimental results, the effects of PVP and PDA in synthesizing PS/Ag composite microspheres were discussed in detail. The silver nanoparticles (Ag NPs) were homogeneously doped into the composite microspheres. The PS/Ag composite microspheres showed the typical surface plasma resonance (SPR) peak of nanosized silver. The effects of silver nanoparticles on the morphology and optical properties of the composite microspheres were studied by TEM and UV-Vis spectra. The experimental approach of this work was simple and easy to operate.

## 2. Experimental Section 

### 2.1. Materials

Styrene (St) was purchased from Shanghai Chemical Reagent Co. (China) and purified by treating with 5% NaOH aqueous solution to remove the inhibitor. Silver nitrate (AgNO_3_, 99%), ammonia hydroxide (25%), poly-vinylpyrrolidone (PVP K30), absolute ethanol (EtOH), 2, 2’-azobisisobutyronitrile (AIBN, 98%), dopamine (DA) and trihydroxymethyl aminomethane (Tris) were also purchased from Shanghai Chemical Reagent Co. (China) and used as received. Deionized water was used for all the experimental processes.

### 2.2. Synthesis of Mono-Dispersed PS Microspheres

Mono-dispersed PS microspheres were synthesized by dispersion polymerization using PVP as the dispersant in the mixture of EtOH and water [12]. In a typical process, St (10.0 g), PVP (5.0 g), AIBN (1.0 g), deionized water (10.0 g) and EtOH (70.0 g) were charged into four-neck round flask equipped with a mechanical stirrer, an N_2_ inlet, a thermometer with a temperature controller, a condenser and a thermostatic water bath. This mixture was deoxygenated by bubbling with nitrogen gas at room temperature for about 30 min, followed by heating to 70 °C. Under a stirring rate of 300 rpm, the polymerization was continued for 6 h.

### 2.3. Preparation of PS-PDA Microspheres

The synthesized PS microspheres were modified with PDA solution (0.02 g dopamine, 0.12 g Tris and 10.0 g deionized water). A 1 mL quantity of as-prepared PS dispersion was immersed in 10 mL PDA solution and reacted for about 24 h under magnetic stirring. The PDA-functionalized PS microspheres were obtained.

### 2.4. Preparation of PS/Ag Composite Microspheres

An appropriate amount of silver nitrate was dissolved into double distilled water to obtain AgNO_3_ (0.12 M) aqueous solution. Subsequently, ammonia (2%) was gradually added into the AgNO_3_ solution until the generated precipitates vanished. A 10 mL quantity of PDA-decorated PS dispersion was added to the 20 mL freshly as-prepared [Ag(NH_3_)_2_]^+^ solution. The mixtures were stirred for 30 min at room temperature to ensure that the [Ag(NH_3_)_2_]^+^ ions were absorbed to the PDA-decorated PS microspheres. Subsequently, the mixtures were heated to 80 °C with stirring for about 60 min. Then, plenty of brown PS/Ag composites were obtained in the solutions. After filtering and fully washing, the as-obtained products were collected and stored in ethanol for further examination. 

### 2.5. Characterization

Transmission electron microscopy (TEM) characterization was performed on a Tecnai 12 electron microscope with an operating voltage of 120 kV. Field emission scanning electron microscopy (FESEM) was carried out with a Hitachi S-4800 scanning electron microscope operating at an acceleration voltage of 15 kV. Elemental mapping images were acquired by energy-dispersive X-ray spectroscopy (EDS) using a Tecnai G2 F30 S-TWIN electron microscope equipped with a scanning transmission electron microscopy (STEM) unit and an Inca Energy 250 detector. Powder X-ray diffraction (XRD) patterns were recorded on a Bruker D and Advance diffractometer with Cu K á radiation. UV-Vis absorption spectra were measured by a Shimadzu UV-2501 spectrophotometer. Surface-enhanced Raman spectroscopy (SERS) was performed with a Renishaw Raman spectrometer.

## 3. Results and Discussion

### 3.1. Synthesis and Morphology of Spheres

**Figure 1:**
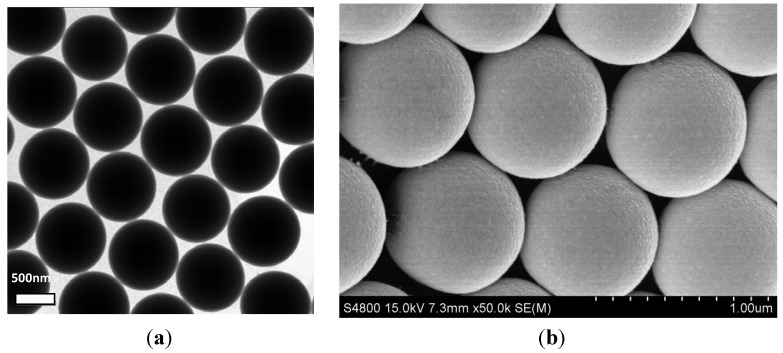

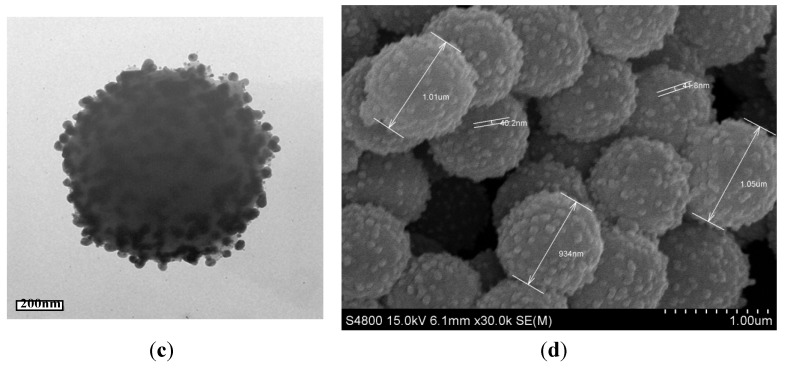
(**a**,**b**) TEM and field emission scanning electron microscopy (FESEM) images of the polystyrene (PS) microspheres with a diameter of 850 nm synthesized by dispersion polymerization; (**c**,**d**) TEM and FESEM images of the PS/Ag composites.

The surface morphologies of the samples before and after being coated with Ag NPs are studied by TEM and FESEM, which are shown in Figure 1. Figure 1a and b present the typical TEM and FESEM images of the bare PS microspheres. It can be seen clearly that the formed PS microspheres are uniform, with smooth surfaces and a diameter of *ca*. 850 nm. Figure 1c and d demonstrate the typical TEM and FESEM images of Ag-coated composites fabricated using 0.12 M [Ag(NH_3_)_2_]^+^ solution. After being coated, the surfaces of the PS beads become rough. The shape of Ag NPs is near-spherical, and all the Ag NPs evenly coat the PS beads. The sizes of the Ag NPs are estimated to be about 40 nm. As shown in Figure 1d, although the surfaces roughened, the monodispersity and spherical shape of PS/Ag composites are mostly preserved. Therefore, mono-dispersed PS microspheres coated with uniform Ag NPs can be successfully synthesized.

**Figure 2:**
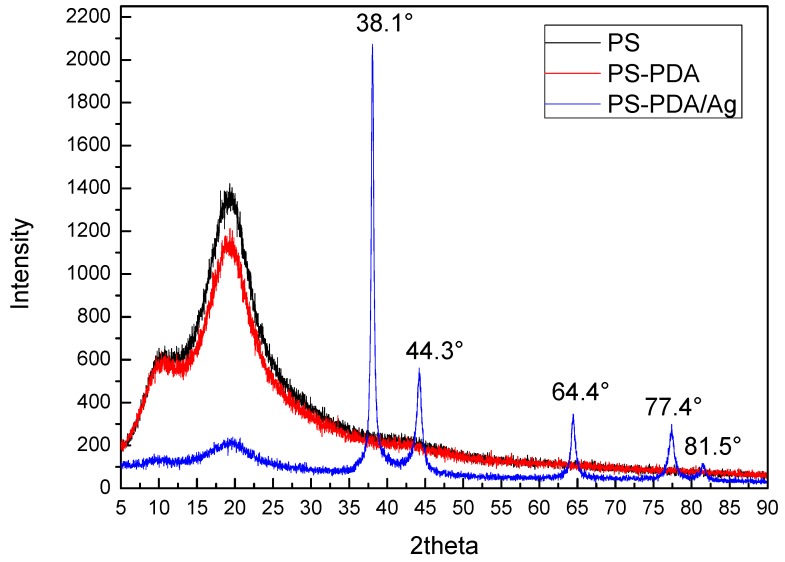
XRD patterns of pristine PS, PS- poly-dopamine (PDA) and PS/Ag composite microspheres.

Figure 2 shows X-ray diffraction patterns of pristine PS, PS-PDA and PS/Ag composites. The strong reflection at 2θ = 20° is assigned to amorphous PS. The typical XRD pattern of PS/Ag exhibits peaks at 2θ angles of 38.1°, 44.3°, 64.4°, 77.4° and 81.5° corresponding to the reflections of the (111), (200), (220), (311) and (222) crystal plane of the face-centered cubic (*fcc*) structure of Ag (Joint Committee on Powder Diffraction Standarda Card 04-0783). It further confirms that silver nanoparticles with crystallinity could be obtained successfully by reducing [Ag(NH_3_)_2_]^+^ ions.

### 3.2. Effects of PVP and PDA on the Formation of PS/Ag Composite Structures

**Figure 3:**
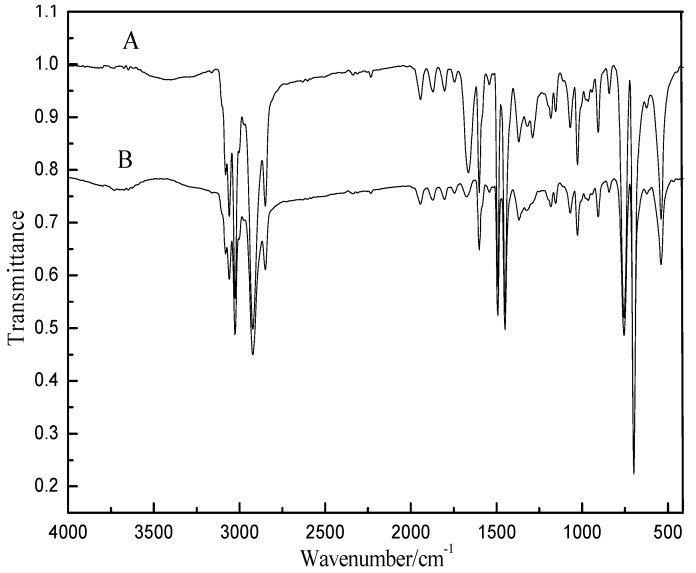
FTIR spectra of (A) unwashed PS microspheres and (B) washed PS microspheres prepared by dispersion polymerization.

In our experiments, PVP first acted as a dispersant to stabilize the formed PS microspheres in the synthesis of PS dispersion. Figure 3 shows the FTIR spectrum of unwashed PS microspheres (A) and washed PS microspheres (B) prepared by dispersion polymerization. As shown in curve A, the absorption band at 1670 cm^−1^ is the typical band of PVP, which decreases significantly, but not completely disappearing in curve B. This is good evidence that the effective stabilizer is PVP containing PS. Some ungrafted PVP adsorbs to the particles and aids in the steric stabilization of the particles during the reaction [13]. A PVP macromolecule in solution, which most likely adopts a pseudorandom coil conformation, may take part in some form of association with the metal atoms, thus increasing the probability of nucleus formation [14]. It has been widely proven by experiments that PVP could be effective in protecting composite spheres from aggregation and in modifying the morphology of composites [15,16,17]. This role of the PS surface in the formation step of silver nanoparticles is similar with that of seed in the heterogeneous nucleation technique [18]. PVP also importantly acted as the protection agent in the reduction of [Ag(NH_3_)_2_]^+^ ions to Ag NPs during our experiments. The inserted higher magnification image (Figure 3) of part of a PS/Ag composite further shows that the narrow size distribution of individual Ag NPs is clear evidence of the separation of nucleation and growth steps caused by the nucleation site role of the PS surface. In addition, immobilization of Ag NPs onto the PS surface acts as a stabilization mechanism for Ag NPs.

**Figure 4:**
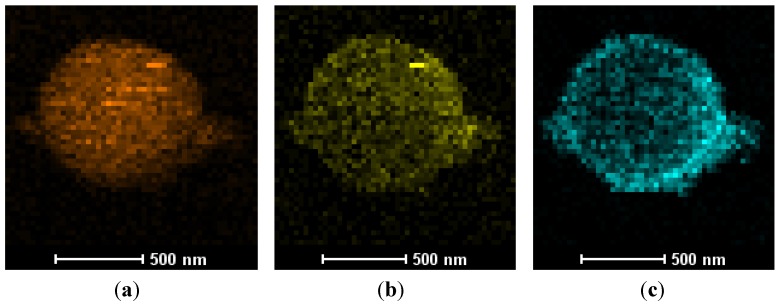
Elemental mapping images of (**a**) N; (**b**) O and (**c**) Ag of a PS/Ag composite microsphere with PDA fabricated using 0.12 M [Ag(NH_3_)_2_]^+^ solution.

To our knowledge, few works have discussed in detail the mechanism of mono-dispersed metal-doped composite microsphere synthesis. PDA is formed by *in situ* spontaneous oxidative polymerization of dopamine and is introduced to the PS microsphere surface. PDA improves the dispersion of hydrophobic PS microspheres in aqueous solution, because of its hydrophilicity [19,20]. More importantly, the metal-binding ability of phenolic hydroxyl groups present in the PDA structure is exploited to absorb [Ag(NH_3_)_2_]^+^ ions onto the PDA-coated PS microsphere surface. The absorbed [Ag(NH_3_)_2_]^+^ ions are reduced to zero-valent silver by the reducibility of PVP and PDA, and silver nuclei are formed on the PS microsphere surfaces. Nuclei are created at the silver ions bound to PDA-coated PS microspheres by the nucleation site role of Ag^+^ ions bound to PDA, similar to the role of seed materials. Finally, Ag NPs are formed on PS microspheres surfaces by the growth of nuclei as the thermal energy supplied to the system increases to a given temperature [21]. Reduced silver species in solution are deposited to silver nuclei immobilized onto the surfaces of PS microspheres, which are attributed to the slower reduction rate of the polyol process than that of the general chemical reduction method using a reducing agent [22]. Elemental mapping analyses on a single PS/Ag composite microsphere are given in Figure 4. The images of nitrogen (Figure 4a) and oxygen (Figure 4b) confirm the presence of PDA on the surface of the PS microsphere. Consequently, this fact means that PDA successfully acts as a chemical protocol between the Ag NPs and the PS surfaces. The image of silver (Figure 4c) indicates the formation of Ag particles loaded onto the PS surfaces.

**Figure 5:**
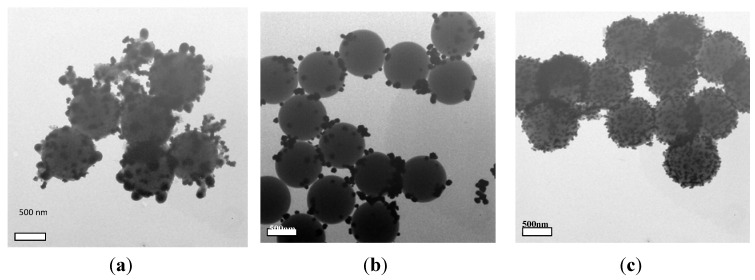
TEM images of the PS/Ag composites (**a**) prepared without PVP and PDA; (**b**) prepared without PDA; and (**c**) prepared with PDA modification.

To confirm the important role of PVP and PDA that could attract [Ag(NH_3_)_2_]^+^ ions onto the surfaces of PS microspheres, control experiments were performed. The other components were experimented on using reducing agent, but without PVP and PDA; a 10 mL washed PS dispersion was added to the 20 mL 0.12 M [Ag(NH**_3_**)**_2_**]**^+^** solution. The other components were experimented on without PDA; just a 10 mL quantity of unwashed PS dispersion was added to the 20 mL 0.12 M [Ag(NH_3_)_2_]^+^ solution. As shown in Figure 5a and b, it can be found that there are Ag NPs dispersed in the system, but they had not been attracted onto PS microspheres to form composite microspheres. Obviously, without the presence of PDA, few [Ag(NH_3_)_2_]^+^ ions are attracted onto the surfaces of PS microspheres; after the formation of zero-valent silver, Ag NPs dispersed in the system separate from PS microspheres. With the appropriate amount of PDA addition, Ag NPs are homogeneously distributed in PS microspheres (Figure 5c). In this system, PDA acts as a nucleation-prompting agent for Ag NPs. It is noticeable that the surfaces of the resultant composite microspheres become rougher.

### 3.3. Concentration Changing of the [Ag(NH_3_)_2_]^+^ Ions

**Figure 6:**
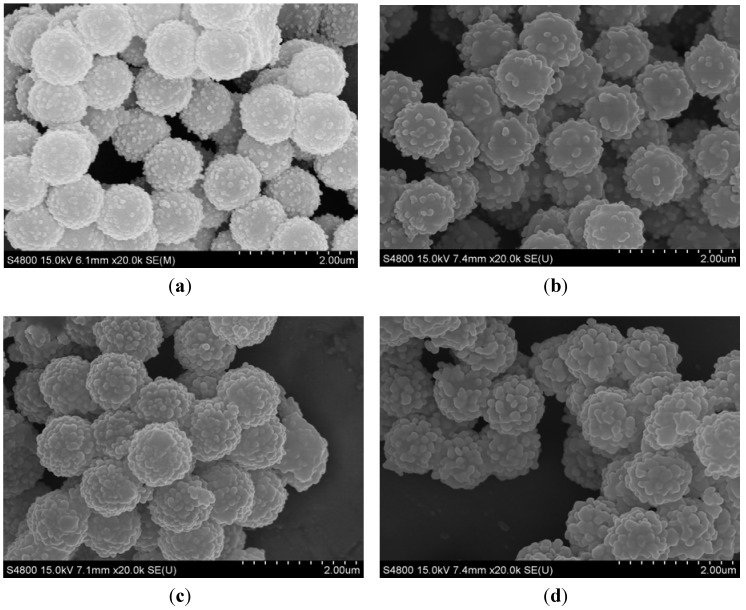
FESEM images of the PS/Ag composites prepared with different concentrations of [Ag(NH_3_)_2_]^+^ ions: (**a**) 0.06 M; (**b**) 0.12 M; (**c**) 0.18 M; and (**d**) 0.24 M.

Based on the *in situ* self-catalytic synthesis method, changing the concentration of the [Ag(NH_3_)_2_]^+^ ions can control the size and coating status of Ag NPs [23]. Figure 6 shows the FESEM images of a series of PS/Ag composites fabricated using different concentrations of [Ag(NH_3_)_2_]^+^ ions ( 0.06 M, 0.12 M, 0.18 M and 0.24 M ). As shown in Figure 6a, the shape of the Ag NPs is near spherical, and an incomplete nanoshell formed because of an insufficient amount of Ag precursor. Upon increasing the concentration of the [Ag(NH_3_)_2_]^+^ ions, the coverage rate of the Ag NPs are elevated, because more [Ag(NH_3_)_2_]^+^ ions were reacted and enhanced the yield of Ag, and the average size of Ag NPs on the PS surface increase from ~20 nm to ~200 nm. However, when the concentration was increased to 0.24 M, the Ag NPs over the PS become nonuniform, and there are many large Ag NPs. With the excess of silver ions in the solution, it is favorable for them to aggregate.

**Figure 7:**
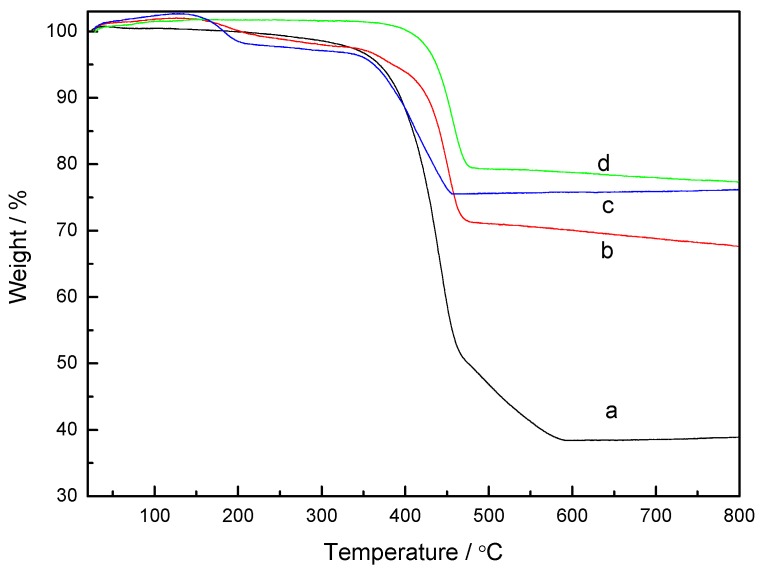
Thermogravimetric Analysis (TGA) curves of the PS/Ag composites prepared with various concentrations of the [Ag(NH_3_)_2_]^+^ ions: (**a**) 0.06 M; (**b**) 0.12 M; (**c**) 0.18 M; and (**d**) 0.24 M.

Thermogravimetric Analysis (TGA) is used to measure the weight percentage of silver in the composites. The pure PS completely decomposes to H_2_, CH_4_ and other gases from 350 to 450 °C, so the residual weight should be that of silver [24]. Figure 7 shows the TGA curves of the samples. It can be seen that the weight loss of the PS/Ag composites took place in the temperature ranges from 400 to 500 °C. According to the TGA curves, the silver contents prepared with various concentrations of the [Ag(NH_3_)_2_]^+^ ions ( 0.06 M, 0.12 M, 0.18 M and 0.24 M ) are found to be 38.92%, 67.65%, 76.16% and 77.33%, respectively. An increase in the silver shell thickness of PS/Ag composites is observed when the concentration of the [Ag(NH_3_)_2_]^+^ ions was increased under the same reaction conditions.

**Figure 8:**
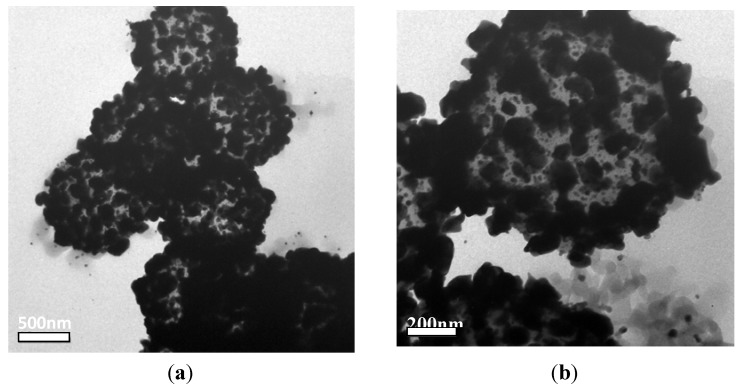
TEM images of the hollow Ag microspheres prepared with 0.12 M [Ag(NH_3_)_2_]^+^ ions. (**a**) magnification 26500; (**b**) magnification 59000.

It is clear that homogenous and complete silver shells form by dissolving the PS cores in tetrahydrofuran (THF). Figure 8 shows the TEM images of hollow silver microspheres. Complete and compact hollow structures formed upon increasing the concentration of the [Ag(NH_3_)_2_]^+^ ions to 0.18 M; the close-packed Ag NPs form a shell. A complete hollow silver microsphere in Figure 8b is the result of the high surface coverage.

### 3.4. Optical Properties of PS/Ag Composites

**Figure 9:**
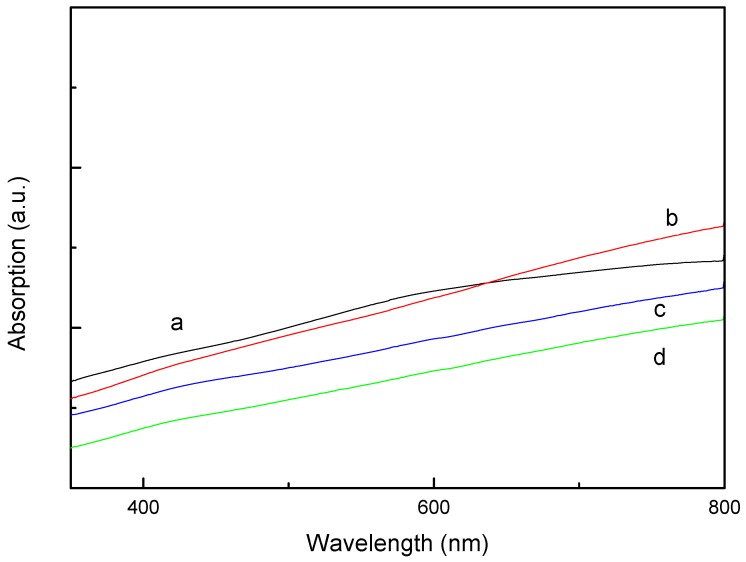
UV-Vis absorption spectra of PS/Ag composites prepared with different concentration of [Ag(NH_3_)_2_]^+^ ions: (**a**) 0.06 M; (**b**) 0.12 M; (**c**) 0.18 M; and (**d**) 0.24 M.

Silver nanoparticles are well known for their surface plasma resonance (SPR) properties, which originate from the collective oscillation of conduction electrons in response to optical excitation [25]. The UV-Vis absorption spectra of the PS/Ag composites are sensitive to size, shape, aggregation state and local environment of the Ag NPs. To investigate the optical properties of the Ag NPs doped in the PS matrix, the samples of PS/Ag composites were diluted with deionized water for measurement of the absorption spectrum. Figure 9 shows the UV-Vis absorption spectra of the PS/Ag composites fabricated using different concentration of the [Ag(NH_3_)_2_]^+^ ions. At relatively low silver concentrations, a broad peak is observed at ~530 nm (curve a), which was assigned to the localized surface plasma resonance (LSPR) of Ag NPs bound to the surface of PS. In addition, there is no SPR peak of the isolated Ag NPs at approximately 420 nm, which confirms that few free Ag NPs appeared in dispersions [17,26]. The position and width of the SPR peak are linked with the size and shape of the metal particles, also connected with its own dielectric constant and that around it. With changing the anisotropy of particles, the peak would vary in the visible and near-infrared spectrum regions [27,28,29]. However, as the silver coverage increased from a low to a high level, the Ag NPs density and the contacting area between Ag NPs and PS supports would increase; so, the absorption spectra of PS/Ag composites are mainly red-shifted and broadening (curves b–d). Curves b–d are not pronounced peaks; the results may be explained as follows. As the silver coverage increases to a high level, the PS/Ag composites mainly show the collective absorption behavior of the Ag NPs. This finding is consistent with the FESEM images shown in Figure 6a–d. The same phenomena had been observed with other works [30,31].

### 3.5. SERS Properties of PS/Ag Composites

**Figure 10:**
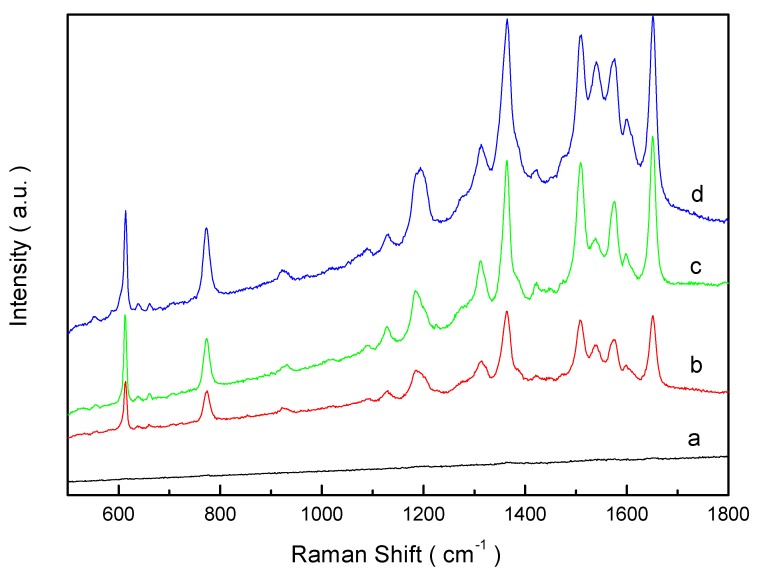
SERS spectra of 1 × 10^−3^ mol/L R6G without PS/Ag composites (**a**) and with PS/Ag composites prepared with different concentration of [Ag(NH_3_)_2_]^+^ ions: (**a**) 0.06 M; (**b**) 0.12 M; (**c**) 0.18 M; and (**d**) 0.24 M.

Surface-enhanced Raman scattering (SERS) is a type of abnormal optical enhanced effect based on the rough surface at the nano-scale, the particle morphology and the granular lumps [32]. Silver is one of the most SERS-active metals. The PS/Ag composites with high Ag NPs coverage were chosen for SERS measurements. The SERS results are shown in Figure 10. As for the 1 × 10^−3^ mol/L Rhodamine 6G (R6G) without PS/Ag composites, the Raman lines for the R6G molecules can be seen from curve a; no peaks appear. However, when the 1 × 10^−3^ mol/L R6G was deposited on the selected PS/Ag composites, the peaks can be seen clearly; almost all the distinctive peaks corresponded to the Raman lines for the R6G molecules. The observed peaks included the ν(C–H) out-of plane bend mode at 774 cm^−1^ and theν(C–C) stretching mode at 1360 cm^−1^, 1508 cm^−1^, which agree well with the literature [33]. With an increase of Ag coverage, the Raman signals are more intensified; this can be seen in curve b–d. As known from the literature, the SERS effects depend strongly on the roughness of the metal nanostructure used as the substrate [34,35].

## 4. Conclusions

In summary, we have demonstrated that mono-dispersed PS/Ag composites could be prepared by a convenient *in situ* reduction of [Ag(NH_3_)_2_]^+^ complex ions. In this method, PVP took both the dispersant and protection agent at the same time. On the basis of experimental results, a polystyrene surface was modified with PDA as a nucleation site during the Ag NP formation period, which is similar to the role of seed materials in the heterogeneous nucleation technique. Therefore, PVP and PDA played an important role in bridging the polystyrene surface and the silver nanoparticles. TEM, FESEM and XRD confirm the formation of PS/Ag composites, which had both the bulk properties of the polystyrene and the surface properties of the Ag NPs. A UV-Vis spectrometer shows that the PS/Ag composites possess good optical properties. Raman spectra indicate that the PS/Ag composites as SERS substrates have excellent SERS performance.

This method provides an alternative approach for the preparation of metal doped polystyrene microspheres. It is also believed that the as-prepared PS/Ag composites have other potential applications, such as catalysis, antibacterial action and the optical detection of macromolecules. Antibody-antigen interaction and DNA hybridization can be dynamically detected from the shift of the SPR peaks [36]. 
